# Oxidized LDLs as Signaling Molecules

**DOI:** 10.3390/antiox10081184

**Published:** 2021-07-26

**Authors:** Jean-Marc Zingg, Adelina Vlad, Roberta Ricciarelli

**Affiliations:** 1 Miller School of Medicine, University of Miami, Miami, FL 33136, USA; 2 Physiology Department, “Carol Davila” UMPh, 020021 Bucharest, Romania; adelina.vlad@umfcd.ro; 3 Department of Experimental Medicine, University of Genoa, 16132 Genoa, Italy; 4 IRCCS Ospedale Policlinico San Martino, 16132 Genoa, Italy

**Keywords:** scavenger receptor CD36, PPARγ, NFκB, Nrf2, PI3K, PKB/Akt, α-tocopherol, vitamin E, atherosclerosis, inflammation, neurodegeneration, stem cells, noncoding RNAs

## Abstract

Levels of oxidized low-density lipoproteins (oxLDLs) are usually low in vivo but can increase whenever the balance between formation and scavenging of free radicals is impaired. Under normal conditions, uptake and degradation represent the physiological cellular response to oxLDL exposure. The uptake of oxLDLs is mediated by cell surface scavenger receptors that may also act as signaling molecules. Under conditions of atherosclerosis, monocytes/macrophages and vascular smooth muscle cells highly exposed to oxLDLs tend to convert to foam cells due to the intracellular accumulation of lipids. Moreover, the atherogenic process is accelerated by the increased expression of the scavenger receptors CD36, SR-BI, LOX-1, and SRA in response to high levels of oxLDL and oxidized lipids. In some respects, the effects of oxLDLs, involving cell proliferation, inflammation, apoptosis, adhesion, migration, senescence, and gene expression, can be seen as an adaptive response to the rise of free radicals in the vascular system. Unlike highly reactive radicals, circulating oxLDLs may signal to cells at more distant sites and possibly trigger a systemic antioxidant defense, thus elevating the role of oxLDLs to that of signaling molecules with physiological relevance.

## 1. Introduction

Oxidative stress occurs during the progression of several diseases. In the cardiovascular system, oxidative stress leads to the formation of oxidized low-density lipoproteins (oxLDLs) with immunogenic and atherogenic properties. In vivo, levels of oxLDLs are usually low but can increase when the balance between the formation of free radicals and their scavenging by antioxidant enzymes and micronutrients is impaired. Under normal conditions, however, a physiological response aimed at removing oxLDLs from circulation is mainly carried out by phagocytes of the reticuloendothelial system, Kupffer cells of the liver, sinusoidal endothelial cells, and macrophages [1]. Under pathologic conditions, these cells become overwhelmed by oxLDLs and the excessive accumulation of lipids transforms them into foam cells, which are a hallmark of atherosclerosis progression [2]. The uptake of oxLDLs is mediated by scavenger receptors that also act as signaling receptors [3]. Indeed, oxLDLs were found to modulate different signal transduction cascades leading to gene expression, apoptosis, adhesion, inflammation, differentiation, migration, and senescence [4,5]. In circulating monocytes, for example, exposure to oxLDLs alters gene expression, stimulates adhesion to the endothelium, and subsequent migration into the intima, where transformation into macrophages and then into foam cells takes place [6]. Circulating oxLDLs can be used as biomarkers since their levels rise in patients with advanced atherosclerosis and also reflect early atherosclerotic changes and metabolic disorders including diabetes and obesity [7].

This review addresses the mechanisms of oxLDL formation, the relevance of oxLDLs in cell signaling and gene expression, and the resulting physio/pathological impact.

## 2. Formation of oxLDLs In Vitro and In Vivo

**Figure 1:**
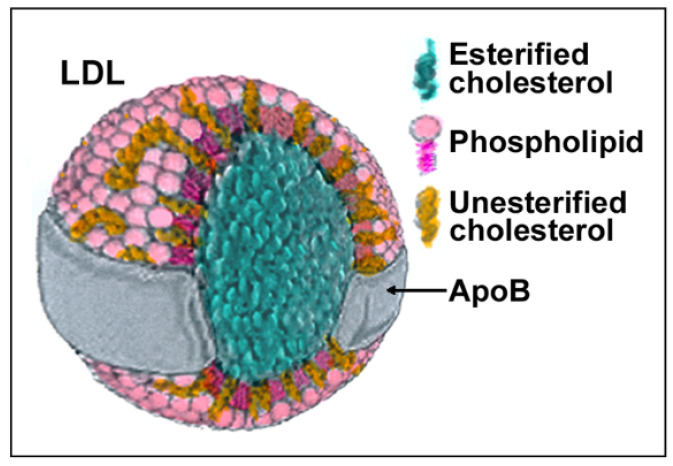
Structure and composition of LDL. Each component of LDLs can be modified by oxidation generating molecules such as oxysterols, oxidized phospholipids, 4-hydroxynonenal (HNE), malondialdehyde (MDA), and modified apoB.

LDLs are composite molecules consisting of a hydrophobic core of polyunsaturated fatty acids and esterified cholesterol surrounded by phospholipids, unesterified cholesterol, and one molecule of apolipoprotein B-100 (apoB) (Figure 1). Based on physiochemical properties, LDLs can be classified into three or four subclasses, including large (LDL I), intermediate (LDL II), small (LDL III), and, in some studies, very small LDL (LDL IV), with the latter being able to easily penetrate the vascular wall and, therefore, be more prone to oxidation. All components of LDL can undergo oxidation [1], generating oxysterols, oxidized phospholipids, and end products of lipid peroxidation such as malondialdehyde (MDA) and 4-hydroxynonenal (HNE) that can be used as markers of LDL oxidation (reviewed in [8]). Adducts between ε-amino groups of lysine residues in apoB and reactive aldehydes such as MDA or HNE can also form, thereby reducing oxLDLs’ affinity for the LDL receptor and increasing their recognition by scavenger receptors [9]. When acting as signaling molecules in distant cells, these different types of oxLDLs may carry information on the severity of lesions and oxidative stress and trigger different responses.

For in vitro experiments, fully (80–100%) oxidized LDLs are usually prepared by exposure to transition metal ions such as Cu^2+^ [10,11] or Fe^2+^ [12]. Incubation with cells producing reactive oxygen species (ROS), or exposure to the myeloperoxidase secreted by activated macrophages, leads to minimally oxidized LDLs, which appear to be better related to the degree of oxidation in vivo [13,14]. Macrophages can oxidize LDL by generating ROS and reactive nitrogen species (RNS) via NADPH-oxidase, lipooxygenase, myeloperoxidase, and nitric oxide synthase (reviewed in [15]). Activated mast cells can also contribute to foam cells and fatty streak formation by stimulating LDL modification and uptake by macrophages [16], by secreting a variety of inflammatory mediators (histamine, leukotrienes, prostaglandins, platelet activating factor) and enzymes (tryptase, chymase, carboxypeptidase and cathepsin G) (reviewed in [17]), likely leading to the weakening and rupture of atherosclerotic plaques [18].

In vivo, LDL oxidation occurs mainly within the subendothelial space of the arterial wall, while other reactions, such as glycation or homocysteinylation, also occur in plasma [19]. In line with this, levels of oxLDLs detectable by immunohistochemistry are higher in arterial lesions and plaques [20], and elevated levels of oxLDL in plasma are thought to originate from the sites of vascular injury [21]. Increased levels of oxLDLs in plasma have been measured during the development of hypertension as well as in uremic and diabetic patients, as evidenced by the presence of autoantibodies against oxLDLs [22,23,24]. Interestingly, in hypercholesterolemic rabbits, the increased expression of the CD36 scavenger receptor in peripheral blood mononuclear cells reflects its levels in aortic lesions and can be used as a diagnostic biomarker for atherosclerosis [25,26].

In addition to professional phagocytes, vascular wall cells, such as endothelial and vascular smooth muscle cells (VSMCs) can also catalyze oxLDL formation both in vivo and in vitro. Two main species of oxLDLs can be distinguished: fully (or extensively) oxidized and minimally oxidized LDLs, the latter being produced in an early atherosclerotic stage and with a longer half-life since they are less efficiently recognized by scavenger receptors [5]. Depending on the concentration and degree of oxidation, oxLDLs may elicit dual cellular responses resulting in the stimulation or inhibition of inflammation, angiogenesis, and survival [5].

Circulating oxLDLs can be used as a marker of oxidative stress [8,27,28,29], which may to some extent be related to the risk of cardiovascular disease. A valid measure of in vivo oxLDL formation is represented by the susceptibility to oxidation of isolated plasma LDLs, as assessed by the lag time of Cu^2+^-induced formation of conjugated dienes [30] that can be spectrophotometrically detected at 234 nm. Another method is to evaluate the acid hydrolysis products of lipoperoxides such as MDA, which reacts with thiobarbituric acid (TBA) to form MDA–TBA adducts. The TBA-reactive substances (TBARS) can be measured spectrophotometrically, fluorometrically, or by high-pressure liquid chromatography. In addition, several immunoassays with antibodies against oxLDLs, MDA-modified LDLs, lysine-substituted LDLs, and oxidized phospholipids have been developed and widely used to measure oxLDLs in biological samples.

## 3. Removal of oxLDLs from the Circulation

The amount of oxLDLs in plasma and tissues is given by the ratio between their formation and the efficiency of their removal. Elimination of oxLDLs from the circulation occurs mainly through the phagocytes of the reticuloendothelial system, including macrophages, dendritic cells, sinusoidal endothelial cells, and Kupffer cells in the liver, or via preformed anti-oxLDL antibodies [1]. In tissues, macrophages and nonprofessional phagocytes remove oxLDLs via internalization by scavenger receptors.

## 4. Atherogenic Effects of oxLDLs and Their Prevention

Oxidized low-density lipoproteins stimulate the expression of endothelial adhesion molecules, have chemotactic effects, and inhibit the migration of macrophages outside the subendothelial space, thus increasing the number of leukocytes and proinflammatory elements involved in atherogenesis [31]. They also stimulate the expression of the scavenger receptors CD36 and SR-A in monocytes, macrophages, and VSMCs. These receptors internalize the oxidized lipoproteins in a specific manner, until foam cells are formed [32]. Moreover, oxLDLs can promote the proliferation of VSMCs, followed by the narrowing of the vascular lumen. For these reasons, strategies to prevent atherosclerosis aim to lower the cholesterol load of lipoproteins and to reduce inflammation and oxidative stress, consequently reducing the atherogenic properties of oxLDLs. When acting as signaling molecules at distant sites (such as the liver and cells of the reticuloendothelial system), the resulting upregulation of scavenger receptors can be seen as a cellular response to stress (e.g., acute injury stress or post-prandial stress [30]) that prepares for the defense and removal of more oxLDLs to come. Accordingly, in monocytes (that normally express low levels of scavenger receptors), oxLDL exposure induces differentiation into macrophages [33,34], whereas in CD36 knockout mice [35] or in human monocytes/macrophages from CD36-deficient patients with a lower capacity to bind and internalize oxLDLs [36,37], decreased NFκB activation after oxLDL stimulation results in a lower expression of inflammatory cytokines [38], suggesting a role of CD36 in oxLDL-stimulated signal transduction.

Given that (1) the rate of oxLDL generation is strictly related to the levels of ROS and RNS and (2) oxLDLs are key determinants of cardiovascular disease [39], the search for effective antioxidant strategies both in vitro and in vivo has been strongly implemented. While LDL oxidation can be chemically prevented in vitro by molecules with antioxidant properties, in vivo antioxidant supplementation has not yet shown clear effects against cardiovascular events. Vitamin E (α-tocopherol), α-tocopheryl quinone/ubiquinone, flavonoids, and β-carotene seem to be the main antioxidant molecules in the hydrophobic core of LDLs, whereas vitamin C (l-ascorbic acid) and uric acid are found in the surrounding plasma (reviewed in [40,41,42]). LDL protection from oxidation also occurs in the subendothelial space, where vitamin E promotes the paraoxonase activity that hydrolyzes and reduces lipid peroxides [43].

### 4.1. Antioxidant Effects of α-Tocopherol

Low plasma levels of α-tocopherol, the major form of vitamin E in plasma, were found to correlate with an increased risk of atherosclerosis [44]. The presence of α-tocopherol in lipoproteins (mainly LDLs) and subendothelial compartments is assumed to play a central role in preventing lipid peroxidation and the consequent vascular damage, as indicated by a number of studies showing that vitamin E prevents the endothelial injury resulting from ROS, oxLDLs, or lipid peroxides [45,46,47,48]. Oral vitamin E supplementation increases the α-tocopherol content in LDLs, the resistance of LDLs to oxidation, and decreases the cytotoxicity of oxLDLs on endothelial cells [49]. In line with this, α-tocopherol and trolox (a more hydrophilic homolog) block early intracellular events such as lipid peroxidation and calcium rise elicited by oxLDLs or linoleic acid hydroperoxide [50,51].

The inflammatory action of oxLDLs in the vascular wall is enhanced by *C. pneumoniae* infection, which leads to cell necrosis that is prevented by vitamin E via inhibition of ROS production and promotion of endothelial cell survival [52]. Furthermore, endothelial cells exposed to oxLDLs exhibit an increased expression of adhesion molecules such as VCAM-1 and ICAM-1, which can be reduced by pretreatment with α-tocopherol [53].

At noncytotoxic concentrations, oxLDLs stimulate VSMCs proliferation and DNA synthesis [54], effects that are expected to be reduced by preventing the oxidation of LDLs via α-tocopherol (reviewed in [55]). Actually, in quiescent VSMC cultures, oxLDLs and lysophosphatidylcholine induced a more than 10-fold increase in DNA synthesis and strongly stimulated cell cycle re-entry. These events were prevented either by α-tocopherol [56,57] or by antioxidant enzymes such as superoxide dismutase and catalase, confirming that the increased cell proliferation in response to oxLDLs was the result of oxidative stress [58,59].

### 4.2. Non-Antioxidant Effects of α-Tocopherol

Alternative mechanisms of α-tocopherol in protection against atherosclerosis have been described, such as modulation of gene expression and cell signaling (reviewed in [60,61,62,63,64]). In vascular endothelial cells, for example, oxLDL/oxysterol-induced necrosis/apoptosis is associated with the generation of intracellular ROS and activation of caspase-3. However, while both of these effects are inhibited by α-tocopherol, β-tocopherol does not affect caspase-3, while maintaining the same efficacy as α-tocopherol against ROS production, thus supporting the idea that α-tocopherol can inhibit caspase-3 in a non-antioxidant manner [65].

In rabbit VSMCs, DNA synthesis is stimulated by oxLDLs, and α-tocopherol limits this effect by both inhibiting oxidation and interfering with the cell signaling elicited by oxidized lipids. In support of this latter mechanism, vitamin E prevented the activation of PKC and the formation of cholesterol-induced atherosclerotic lesions, while the powerful antioxidant probucol was not effective. In that study, a group of animals received a vitamin-E-poor diet containing 2% cholesterol, while another group received the same diet plus vitamin E or probucol intramuscularly for 4 weeks. The obtained results showed that the protective effect of vitamin E against atherosclerosis was not mimicked by probucol and, therefore, may not be due to the antioxidant properties of vitamin E [66].

Another important piece of evidence related to the non-antioxidant effects of vitamin E concerns its ability to lower the expression of the scavenger receptor CD36. In VSMCs, this effect reduced oxLDL uptake and oxLDL-mediated signal transduction, possibly resulting in decreased hyperplasia [34,67]. Signaling in response to oxLDLs is primarily related to the activation of protein kinase C (PKC) and protein kinase B (PKB/Akt), both of which are inhibited by vitamin E. In murine macrophages, activation of PKC by oxLDLs leads to stimulation of peroxisome proliferator receptor gamma (PPARγ) and CD36 expression [68]. In THP-1-derived macrophages, the increased expression of CD36 and SR-A, and the consequent cholesterol uptake and foam cell formation, occurs following activation of PKCδ, PI3K/PKB, and ERK by oxLDLs [69]. Vitamin E inhibits PKCα in VSMCs [70,71,72], but in other cell types, it appears to inhibit PKCδ as well [73]. In monocytes, inhibition of PKC by vitamin E affects superoxide production by preventing the assembly of NADPH-oxidase; again, an effect that has not been mimicked by vitamin E analogs with similar antioxidant potential [74]. Among others, cellular events modulated by the vitamin include proliferation, migration, and adhesion [75,76].

### 4.3. Prooxidant Effects of α-Tocopherol

It has been reported that lipid peroxidation of LDL can be significantly accelerated by increasing their vitamin E content. Peroxidation is assumed to be propagated by the α-tocopheroxy radical of vitamin E [31,77], but the in vivo relevance of pro-oxidant reactions of α-tocopherol has yet to be clarified.

## 5. The oxLDL Signaling

The scavenger receptors LOX-1, SR-A, SR-B1, and CD36 are at the forefront of the response to oxLDLs, although they recognize lipoproteins modified not only by oxidation but also by glycation, alkylation, and nitration and internalize them promoting their removal and degradation [3,78,79,80]. Professional phagocytes of the reticuloendothelial system including macrophages, dendritic cells, and Kupffer cells of the liver are primarily responsible for the scavenger receptor-mediated removal of oxLDLs. LOX-1, in particular, is the foremost receptor recognizing oxLDLs in the cells of the reticuloendothelial system. Many other cell types, however, express scavenger receptors able to internalize oxLDLs, such as VSMCs, endothelial cells, neuronal cells, and keratinocytes. Though these “nonprofessional” phagocytes mediate the local clearance of oxLDLs, an excessive uptake without an efficient degradation machinery may lead to cellular deregulation, apoptosis, and formation of foam cells [81]. As described below, scavenger receptors can mediate the oxLDL signal through two main pathways that can actually occur in combination: (1) by internalization of oxLDL and release of their content with signaling functions and (2) by themselves acting as signaling receptors, often together with co-receptors and intracellular proteins.

### 5.1. Signaling by the Oxidized Lipid Content of oxLDLs

Uncontrolled uptake of oxLDLs and lipids ultimately converts monocytes/macrophages and VSMCs to foam cells. In this process, scavenger receptors play a critical role due to their ability to internalize oxLDLs and transport lipids and cholesterol in and out of cells [81,82]. In fact, scavenger receptors are highly expressed at the atherosclerotic lesion, where macrophages show increased SR-AI/II, SR-BI, and CD36 levels [83,84,85], whereas VSMCs and endothelial cells overexpress CD36 and LOX-1 [67,86,87,88,89].

**Figure 2:**
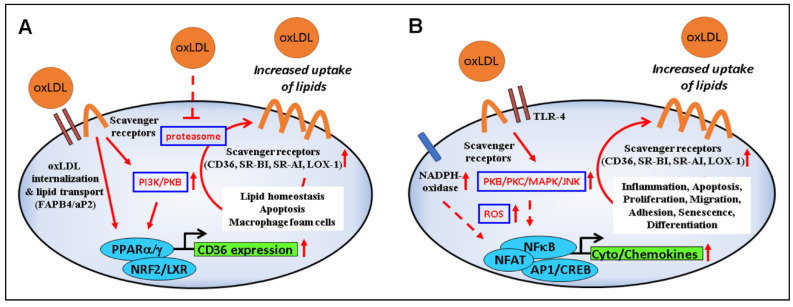
Modulation of signaling and gene expression by oxLDL. (**A**) OxLDL-induced upregulation of scavenger receptors via PI3K/PKB/PPARγ and proteasome inhibition leads to increased internalization of oxLDLs and their load (lipids, oxidized lipids, and products of lipid peroxidation such as HNE), with consequences on lipid homeostasis, foam cell formation, and cell viability. (**B**) Binding of oxLDLs to scavenger receptors and TLR4 activates the PKB–JNK pathway which, in turn, stimulates NADPH oxidase activity, further supporting ROS production, inflammation, apoptosis, proliferation, migration, cell differentiation, and senescence.

The increased expression of CD36 in oxLDL-treated cells is mediated by PPARγ, PKB/Akt, and NF-E2-related factor (Nrf2) (Figure 2A) [33,90,91], although PKC has also been involved in the process [68]. On the other hand, cholesterol and cholesterol acetate induce CD36 through the activation of sterol regulatory binding proteins (SREBP-1/2) [92,93]. After internalization by target cells, oxLDL degradation products (oxysterols, oxidized phospholipids, HNE, etc.) interfere with mitogen-activated protein kinases (MAPKs), as well as the survival-associated PI3K/Akt pathway and transcription factors such as AP-1 and PPARγ [94], which can lead to signals implicated in vascular cell apoptosis and plaque instability, adhesion of circulating blood cells, foam cell formation, and fibrogenesis [95,96].

Oxidized LDLs also reduce the proteasome activity [97], possibly explaining the increased levels of ubiquitinated proteins in unstable atherosclerotic plaques [98,99]. Inhibition of the proteasome is associated with an increased expression of CD36 [100,101,102]; however, it is still unclear whether vitamin E, which is known to reduce CD36 levels and oxLDL uptake, can restore the cellular proteasome activity [101,102,103,104]. Proteasome inhibition may result from the formation of adducts between HNE, which is increased in vitamin E deficiency [105], and one specific proteasomal subunit (i.e., Rpt4), leading to a biphasic response to oxLDLs, characterized by an early transient activation followed by inhibition [97,106]. Accordingly, CD36 is upregulated by HNE and some specific lipids through the involvement of Nrf2, a transcription factor relevant to establish the senescent phenotype [91,107,108,109,110]. In endothelial cells, senescence is induced by HNE of macrophage-derived foam cells [111], and atherosclerotic lesions in LDL receptor knockout mice show an increased number of senescent cells contributing to inflammation and plaque instability [112]. Of note, the oxLDL-induced cellular senescence can be counteracted by vitamin E through downregulation of CD36 expression, ROS scavenging, and inhibition of NADPH oxidase membrane translocation [113]. A role for CD36 in cellular senescence has also recently been described in relation to the senescence-associated secretory phenotype (SASP) and lysosomal β-galactosidase (SA-β-gal) [114,115].

### 5.2. Signaling by oxLDL-Activated Scavenger Receptors

Binding of oxLDLs to scavenger receptors can trigger a number of intracellular events that depend on the type of cell and scavenger receptor involved (Figure 2B) (reviewed in [3,116,117]). Here, we highlight the signaling related to CD36, which has been extensively investigated.

Several events, such as inflammation, angiogenesis, phagocytosis, and energy homeostasis have been related to the activity of CD36 [118]. This scavenger receptor modulates the uptake of a number of lipids (anionic phospholipids, long-chain fatty acids, diacylglycerides, vitamin E, vitamin D, vitamin A), but it also mediates their effects by acting as a co-receptor (e.g., with Toll-like receptor 4/6) and/or allowing the lipid transfer to other receptors, or via a short intracellular domain that interacts with kinases such as Lyn, Fyn, and Lck [117]. In addition to oxLDLs, CD36 has numerous other ligands with important physiological functions ranging from the detection of flavors in olfactory epithelial cells to the phagocytosis of pathogens and to the regulation of lipid uptake and storage [117,119,120]. Of relevance may be the competition of these ligands with oxLDLs for CD36 binding, the ligand-induced downregulation of CD36 on the cell surface [34,67,121,122,123,124], and the CD36-mediated induction of endoplasmic reticulum stress by oxLDLs in various cell types [125].

### 5.3. Signaling of oxLDL to Cells Close to the Atheroma

In monocytes/macrophages and VSMCs nearby the atherosclerotic lesion, oxLDLs can increase the expression of scavenger receptors as a result of a positive feedback loop [85,86,90,126]. Experiments performed in THP-1-derived macrophages demonstrated that exposure to oxLDLs induces CD36 expression [127,128] that, in turn, sustains the uptake of oxLDLs [128,129]. Consequently, phenotypic polarization of macrophages, as reflected by the gene expression pattern, is drastically modified [130]. In line with this, human monocytes/macrophages from CD36-deficient patients showed a lower capacity to bind and internalize oxLDLs [36,37], together with a decreased NFκB activation and a lower expression of inflammatory cytokines after oxLDL stimulation [38]. In addition, in mice, disruption of the CD36 gene, or transplantation of stem cells in which CD36 had been knocked down, prevented the development of atherosclerotic lesions [35,131]. In mouse bone marrow-derived macrophages, anti-apoptotic and pro-survival effects of oxLDLs have also been described [132].

In VSMCs, oxLDLs activate PKB/Akt inducing cell proliferation [133]. Moreover, low concentrations of oxLDLs are capable of triggering the transition of primary VSMCs to a proinflammatory phenotype characterized by changes in the expression of contractile proteins and myocardin. In particular, these effects were abolished by the downregulation of the multifunctional urokinase receptor (uPAR) which, in response to oxLDLs, associates with CD36 or TLR4 to form a receptor cluster capable of mediating changes in VSMC protein expression [134].

In endothelial cells and THP-1 monocytes, oxLDLs upregulate vascular endothelial growth factor (VEGF), a critical angiogenic factor for atherosclerosis, as it induces endothelial cell proliferation, vascular permeability, and macrophage migration. The expression of VEGF implies the activation of PPARγ [135], which is elicited by oxidized components of oxLDLs such as 9-hydroxyoctadecadienoic acid (9-HODE), 13-hydroxyoctadecadienoic acid (13-HODE), 15-deoxy-delta12,14 prostaglandin J2 (15d-PGJ2), and retinoic acid (RA). Activation of PPARγ also increases the expression of the adipocyte lipid-binding protein (ALBP/aP2) that serves as a lipid shuttle, delivering hydrophobic fatty acids to their targets. In THP-1 macrophages, oxLDL-induced ALBP/aP2 gene expression requires activation of both NFκB and PKC signaling pathways [135,136,137].

Platelets have been shown to internalize oxLDLs, followed by lowered eNOS activity and enhanced human platelet aggregation, whereas LOX-1 antibody administration decreased arterial thrombus formation in an in vitro setting [138].

The removal of oxidized lipids operated by the lymphatic vessels seems to play a significant anti-atherogenic role, although the precise mechanism remains to be identified. A very recent study demonstrated that, in human atherosclerotic arteries, oxLDLs especially accumulate in the adventitial layer, which is particularly rich in lymphatic vessels [139]. Treatment of human lymphatic endothelial cells with oxLDLs inhibited the in vitro tube formation, an effect that was prevented by siRNA-mediated knockdown of CD36 [139].

Although the anti-angiogenic actions of oxLDLs are quite well known, a number of studies provide the opposite evidence, indicating a stimulatory effect of low oxLDL concentrations on the formation of new vessels. Incubation of human umbilical vein endothelial cells (HUVECs) with oxLDLs activates LOX-1 expression leading to the upregulation of adhesion molecules, inflammatory proteins, tissue factors, and remodeling proteins that promote angiogenesis [140]. Moreover, Dandapat and colleagues demonstrated that oxLDLs (<5 μg/mL) stimulate capillary tube formation from endothelial cells via LOX-1-dependent activation of the NADPH oxidase/MAPK/NFκB pathway [141]. Despite the beneficial effects in ischemic tissues, angiogenesis sustains plaque development in early stages of atherosclerosis [142] and can induce plaque vulnerability by hemorrhagic events in the microvasculature of advanced atheroma [143].

### 5.4. Signaling of oxLDLs to Distant Tissues

Unlike the vascular wall, where the overexpression of scavenger receptors favors the atherosclerotic process, in nonvascular cells of distant tissues it could help lower plasma lipids, as CD36/FAT mediates the uptake of long-chain fatty acids [144,145]. However, although several independent studies have reported reduced lipid levels in the plasma of transgenic mice overexpressing CD36 [37,146,147,148,149,150,151], a larger analysis clearly suggests that CD36 and oxLDLs form a pathogenic pair in different tissues.

In liver, the oxidized lipid load of oxLDLs contributes to nonalcoholic steatohepatitis (NASH) by increasing lipid accumulation (steatosis) along with inflammation (hepatitis) [152]. Higher levels of CD36 in the female liver, compared to that in the male liver, may contribute to gender differences in susceptibility to diseases such as hyperlipidemia and insulin resistance [153]. Interestingly, the liver x receptor (LXR), which responds to cholesterol and oxysterols, upregulates the alpha-tocopherol transfer protein (α-TTP), suggesting that increased oxLDLs and oxidative stress may signal the need for increased vitamin E retention [154,155,156]. Increased liver vitamin E levels may also prevent liver fibrosis mediated by lipid peroxidation products (e.g., HNE) after carbon tetrachloride exposure [157].

In adipose tissue, CD36 acts as a fatty acid transporter, but it can also mediate endocytosis of oxLDLs as in professional phagocytes [158]. Indeed, in vivo studies performed in mice revealed that oxLDL can induce a CD36-dependent inflammatory paracrine loop between adipocytes and their associated macrophages [159]. In cultured murine adipocytes, oxLDLs inhibited the expression of leptin, an effect that was prevented by anti-CD36 antibodies and the ROS inhibitor N-acetylcysteine [160]; moreover, exposure to oxLDLs reduced the recruitment of glucose transporter 4 (GLUT4) to the plasma membrane, resulting in impaired insulin signaling [161]. Data obtained with human visceral fat from nonobese subjects strengthened this evidence, indicating that adipocytes exposed to oxLDLs assume an inflammatory phenotype with decreased leptin secretion, low insulin-induced glucose uptake, and altered expression of genes involved in apoptosis, autophagy, necrosis, and mitophagy [162].

In the heart, oxLDLs have been reported to reduce GLUT4 expression [163]. It is worth noting that, in addition to reduced insulin signaling, cardiomyocytes showed intracellular accumulation of ceramides and Ca^2+^, irregular electrical activity, and rapid ATP depletion [164,165,166].

A wealth of data also links oxLDLs to pancreatic beta cell damage, a process that can be neutralized by HDLs, VLDLs, and antioxidants [167]. Studies performed in vitro, for example, demonstrated that oxLDL (but not native LDL) treatment induces a specific signaling cascade resulting in impaired insulin production and increased apoptosis [168,169].

In the kidney, glomerular and tubulointerstitial lesions have been related to the effects of oxLDLs on podocytes and mesangial and tubular cells. Oxidized LDLs promote cell proliferation [170], adhesion of monocytes to mesangial cells, and production of matrix components in the mesangium [171], ultimately leading to glomerulosclerosis. LDLs incubated with human mesangial cells undergo peroxidation and stimulate collagen mRNA expression that can be reduced by treatment with vitamin E or anti-oxLDL antibodies [172]. Moreover, in cultured human podocytes, oxLDLs induce apoptosis and decrease the expression of nephrin, a slit diaphragm-associated protein, resulting in cell retraction and increased diffusion of albumin through their monolayer [173]. Notably, the main receptor responsible for the uptake of oxLDLs in podocytes is CXCL16 [174], whereas CD36 is more involved at the mesangial and tubular level.

A role for oxLDLs in the pathophysiology of bone disorders has also been suggested. A recent study conducted on human mesenchymal stem cells reported that exposure to oxLDLs inhibits osteoblast differentiation. As an underlying mechanism, the authors proposed that oxLDLs interfere with the canonical Wnt signaling pathway in a CD36-dependent manner, leading to the inhibition of osteoblastogenesis [175]. In fact, a study published ten years earlier had led to similar conclusions, showing that oxLDLs (but not native LDLs) inhibit stromal cell osteoblastic differentiation and stimulate adipogenesis, supporting the “lipid hypothesis of osteoporosis”. These events were found to involve PPARα and PPARγ [176].

In both breast and ovarian cancer, patients showed increased oxLDL levels compared to controls, suggesting a possible role of oxLDLs in the process of malignancy [125,177]. Certainly, in human breast mammary epithelial cells, oxLDLs triggered the upregulation of proliferative and pro-inflammatory signaling. Interestingly, as in vascular cells, LOX-1 and CD36 scavenger receptors, NADPH oxidase, lipoxygenases-12 and -15, and cytoplasmic (but not mitochondrial) SOD were upregulated by oxLDLs. Furthermore, oxLDLs stimulated p44/42 MAPK, PI3K and Akt, while intracellular PTEN was found to decrease. The effect on PTEN was attributed to the induction of hsa-miR-2, which leads to the activation of the PI3K/Akt pathway [178].

In the sub-retinal pigment epithelium (sub-RPE) space of the macula, deposits of oxLDLs are considered contributors to the onset and development of age-related macular degeneration (AMD). In fact, a recent study found that, in a human-derived RPE cell line, exposure to oxLDLs induces a rapid response involving over 400 genes, including antioxidant and detoxifying genes regulated by Nrf2 and aryl hydrocarbon receptor [179]. However, no correlation was observed between serum levels of oxLDLs and AMD, indicating that RPE is more likely to be affected by locally formed oxLDLs [180].

In the brain, an increase in oxLDLs has been associated with hyperlipidemia and impaired blood–brain barrier, which could contribute to neurodegenerative events and vascular dementia [181,182]. In this context, increased serum oxLDL levels have been detected in Alzheimer’s patients and have been suggested as biomarkers for the disease (reviewed in [125]). In cultured embryonic neurons, astrocytes, and microglia, both oxLDLs and oxHDLs induced lethal oxidative stress that was amplified by amyloid-beta or glutamate [183,184].

### 5.5. Signaling of oxLDLs to Stem Cells

Most diseases in which an increased plasma oxLDL level is a certified biomarker (e.g., coronary heart disease, metabolic syndrome, and systemic autoimmune disorders) are often associated with low levels of circulating endothelial progenitor cells (EPCs). This observation was confirmed by a series of experimental tests on various types of progenitor cells that are distributed throughout the body. In vitro exposure of EPCs to oxLDLs suppressed survival, proliferation, migration, and vasculogenesis [185]. Nevertheless, more recent studies indicated the existence of a dose-dependent biphasic effect of oxLDLs on human EPC tube formation, both in vitro and in vivo [186]. The toxicity of oxLDLs was related to activation of LOX-1 and MAPKs, hyper production of ROS, inhibition of the PI3K/Akt pathway, and downregulation of eNOS (reviewed in [187]). Many of these deleterious effects were prevented by statin administration, but hyperglycemia has shown synergistic action with oxLDLs on survival and EPC function [187].

Under experimental conditions, mesenchymal stem cells (MSCs) are the preferred source for stem cell transplant therapy in various diseases [188] due to their ability to differentiate into different cell types. In mouse MSCs, properties important for engraftment (e.g., proliferation, migration, and adhesion) were stimulated by oxLDLs through activation of LOX-1 and expression of the monocyte-1 chemoattractant protein (MCP-1) [189,190]. In addition, oxLDLs were able to induce cardiac differentiation of cultured MSCs via activation of ERK1/2 signaling pathway [191].

Interestingly, MSCs interfere with a number of atherogenic and cardiotoxic events induced by oxLDLs. In human and mouse endothelial cell cultures, for example, the presence of human MSCs reversed the effects of oxLDLs by restoring Akt/eNOS activity, while MSC-infused apoE^-/-^ mice showed lower endothelial dysfunction and reduced plaque formation [186]. In lipopolysaccharide-injured cardiomyocytes, exosomes derived from MSCs inhibited LOX-1 expression and significantly reduced apoptosis and autophagic response [192].

In neurodegenerative diseases related to oxidative stress and high plasma levels of oxLDLs, a decline in neuronal progenitor cells (NPCs) results in a reduced capacity for neural regeneration [193]. In line with this, in vitro administration of oxLDLs exerts suppressive influences on NPC differentiation by inhibiting activation of PKB/Akt and CREB (cAMP response element binding protein) [194].

### 5.6. Signaling of oxLDL to Noncoding RNAs

Recent studies are testing the hypothesis that the effects of oxLDLs on signaling and gene expression may involve a series of noncoding RNAs (ncRNAs). Indeed, oxLDLs modulate important microRNAs (miRs) implicated in vascular cell homeostasis and related to endothelial function, inflammation, and lipid uptake [195]. In VSMCs, for example, the oxLDL-induced migration is orchestrated through epigenetic modification of the MMP2/MMP-9 genes following miR-29b upregulation [196]. Furthermore, oxLDLs interfere with signal transduction in VSMCs and macrophages by reducing the expression of miR-let-7g through stimulation of the transcription factor Oct-1 [195,196].

Interestingly, oxLDLs have been suggested as a common pathogenic factor of atherosclerosis and tumors [197], indicating miR-210A upregulation as a likely mechanism underlying this association [198], although other miRs, such as miR-21, have been involved in oxLDL-related carcinogenesis [178]. Additionally, miR-21, among other effects, appears to contribute, together with miR-9, to the alteration of insulin secretion induced by oxLDLs in pancreatic cells, an effect that was prevented by co-incubation with HDL [199].

On the other hand, some miRs have shown vascular protective effects, most of which offer valuable perspectives for therapy. Upregulation of miR-155 after oxLDL treatment, for example, reduces the secretion of TNF-α, IL-6, and IL-8 [200,201], lowers the expression of adhesion molecules and chemotactic factors in macrophages and dendritic cells, and inhibits scavenger receptors and lipid uptake [202,203]. Similarly, in oxLDL-stimulated macrophages, exogenous miR-146a lowered the cholesterol burden and the release of inflammatory factors by inhibiting the activation of TLR4-dependent signaling [204]. Moreover, miRs shuttled by extracellular vesicles have been shown to contribute to the anti-atherosclerotic effects of MSCs. In particular, miR-221 delivered in this way could reduce atherosclerotic plaque formation in mice [205], while miR-512-3p protected endothelial cells against oxLDLs by targeting the Kelch-like ECH-associated protein 1 (Keap1) [206]. Finally, in a human brain microvascular endothelial cell line (HBMEC), the pathogenic effects of oxLDLs on proliferation, migration, apoptosis, and ROS and NO production were counteracted by miR-25-5p overexpression [207,208] and miR155 knockdown [209].

One particular type of ncRNA is circular RNA (circRNA), so named because of its unique covalent closed-loop structure. CircRNAs are stable and rich in miR-binding sites that serve as miR sponges or competitive endogenous RNAs. Currently, it has been shown that they are able to modulate the proliferation, migration, inflammatory response, and apoptosis of oxLDL-stimulated endothelial cells [210,211,212], macrophages [213,214], and VSMCs [215,216].

Large numbers of long noncoding RNAs (lncRNAs) have also been identified in oxLDL-treated monocytes, endothelial cells, and VSMCs (reviewed in [217]), and some of them interfere with cholesterol efflux and cholesterol/oxLDL-mediated inflammation [218,219].

## 6. Concluding Remarks

**Figure 3:**
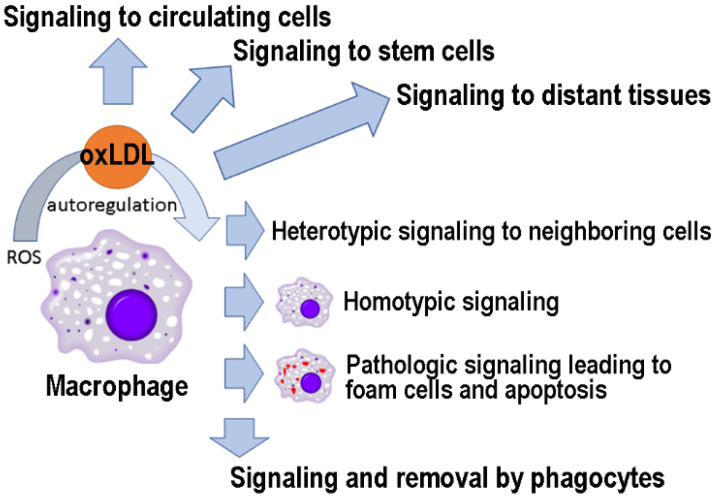
OxLDLs as signaling molecules to cells in different tissues.

LDL oxidation occurs mainly locally, at the site of oxidative stress, in response to free radicals with a short half-life and limited range of action. However, as described here (Figure 3), oxLDLs can function as signaling molecules at distant sites and are likely to play an important role in maintaining the systemic response to oxidative damage. Furthermore, oxLDL-induced changes in the interactome network could serve as biomarkers to follow the physio/pathological relevance of these signaling events [175,220]. In conclusion, as has happened with other “bad” molecules (e.g., amyloid-beta) [221], future studies may reveal that oxLDLs have hormetic effects, being useful in low concentrations and harmful in high quantities.
